# Frequency of the *TP53* R337H variant in sporadic breast cancer and its impact on genomic instability

**DOI:** 10.1038/s41598-020-73282-y

**Published:** 2020-10-06

**Authors:** Carolina Mathias, Stéfanne Bortoletto, Ariana Centa, Heloisa Komechen, Rubens S. Lima, Aline S. Fonseca, Ana Paula Sebastião, Cícero A. Urban, Emerson W. S. Soares, Carolina Prando, Bonald C. Figueiredo, Iglenir J. Cavalli, Luciane R. Cavalli, Enilze M. F. S. Ribeiro

**Affiliations:** 1 Graduate Program of Genetics, Department of Genetics, Federal University of Paraná, Curitiba, Paraná 81531980 Brazil; 2 Faculdades Pequeno Príncipe, Instituto de Pesquisa Pelé Pequeno Príncipe, Curitiba, Paraná 80250060 Brazil; 3 Breast Disease Center, Hospital Nossa Senhora das Graças, Curitiba, Paraná 80810040 Brazil; 4 Department of Pathology, Hospital de Clínicas, Federal University of Paraná, Curitiba, Paraná 81531980 Brazil; 5 Service of Pathology, Hospital Nossa Senhora das Graças, Curitiba, Paraná 80810040 Brazil; 6 União Oeste Paranaense de Estudos E Combate Ao Câncer, Cascavel, Paraná 85806300 Brazil; 7 Lombardi Comprehensive Cancer Center, Georgetown University, Washington, DC 20007 USA

**Keywords:** Breast cancer, Cancer genetics, Cancer genomics

## Abstract

The R337H is a *TP53* germline pathogenic variant that has been associated with several types of cancers, including breast cancer. Our main objective was to determine the frequency of the R337H variant in sporadic breast cancer patients from Paraná state, South Brazil, its association with prognosis and its impact in genomic instability. The genotyping of 805 breast cancer tissues revealed a genotypic and allelic frequency of the R337H variant of 2.36% and 1.18%, respectively. In these R337H+ cases a lower mean age at diagnosis was observed when compared to the R337H-cases. Array-CGH analysis showed that R337H+ patients presented a higher number of copy number alterations (CNAs), compared to the R337H−. These CNAs affected genes and miRNAs that regulate critical cancer signaling pathways; a number of these genes were associated with survival after querying the KMplot database. Furthermore, homozygous (R337H+/R337H+) fibroblasts presented increased levels of copy number variants when compared to heterozygous or R337H− cells. In conclusion, the R337H variant may contribute to 2.36% of the breast cancer cases without family cancer history in Paraná. Among other mechanisms, R337H increases the level of genomic instability, as evidenced by a higher number of CNAs in the R337H+ cases compared to the R337H−.

## Introduction

The R337H is a germline pathogenic variant at codon 337 of exon 10 of the *TP53* gene, which occurs outside the DNA binding region in the dimerization region of the p53 protein. The exchange of arginine by a histidine (R337H CGC → CAC) characterizes the variant, which affects the structure (tetramer formation) of the protein, leading to an unstable molecule with the eventual loss of function^1^.

The R337H was first reported in the southern region of Brazil as a germline variant in pediatric patients with adrenocortical tumors (ACTs), where it was identified in 95% of the cases^2^. It was later characterized as a low penetrance *TP53* variant^3,4^. Large population studies evaluating the presence of *TP53* R337H in the state of Paraná in southern Brazil revealed its presence in 0.27%^5^ and 0.306%^4^ of 214,087 tested newborns. These clustered populations and others reported with the *TP53* R337H variant can be attributed to a founder effect^6^.

Families carrying the germline *TP53* R337H variant present a higher but variable incidence of cancer^3–5,7–11^. ACTs and choroid plexus carcinoma are the most prevalent types of cancer among children in these families, while breast, stomach, and brain cancers are the most common in the higher age groups^7,11^.

A study conducted with tumor samples from the Brazilian states of São Paulo and Rio Grande do Sul reported an 8.6% (70/815) frequency of the *TP53* R337H variant among women with breast cancer without family history^12^. These and other authors have found breast cancer cases and other tumors positive for R337H associated with Li–Fraumeni syndrome (LFS) or Li–Fraumeni-like syndrome, hereditary and non-hereditary breast cancer^13–20^, and/or asymptomatic high risk patients^21^. Despite the accumulating and compelling evidence of the association between R337H and breast cancer, few studies have investigated the cellular phenotype and/or the genomic “consequences” of the R337H variant in breast cancer cells^22–24^.

Genomic instability is a hallmark of cancer that occurs in virtually all types of cancer. Genomic instability is essential for tumor progression^25,26^. The instability can be evident as several DNA and/or chromosome alterations, including gene amplifications, chromosome rearrangements, and changes in DNA copy number.

The main objective of this study was to determine the frequency of the *TP53* R337H variant in patients diagnosed with sporadic breast cancer from the state of Paraná and its association with clinical and histopathological parameters to assess its potential prognostic value in this population. To our knowledge, no studies have assessed the direct impact of the R337H variant on the genomic instability of these tumors. We also aimed to determine the patterns of the genome-wide copy number alterations (CNAs) in these cases and their corresponding effects on signaling and functional cellular pathways. In addition, to verify whether R337H+/R337H+ and Wt/R337H+ cells contribute equally to genomic instability, we generated normal cell cultures of fibroblasts from individuals with homozygous and heterozygous *TP53* R337H variants exposed to a DNA damage agent and evaluated the patterns of their copy number variations (CNVs).

## Results

### *TP53* R337H variant frequency

Nineteen patients were identified by real-time PCR as heterozygous carriers of *TP53* R337H among 805 women with sporadic breast cancer. All 19 positive patients had their genotypes confirmed by Sanger sequencing. The genotypic and allelic frequencies were 2.36% and 1.18%, respectively.

### *TP53* R337H status and clinical-histopathological parameters, and survival

The breast cancer patients were subdivided into two groups according to the R337H variant status. One group of patients harbored the R337H variant (R337H+; n = 19). The other group were non-carriers (R337H−; n = 50). The non-carriers were selected based on the criteria described in the Materials and Methods. The R337H+ group had a significantly lower mean age at diagnosis compared to the R337H− group (47.88 ± 11.56 and 58.52 ± 15.18 years, respectively; Student’s *t*-test, *t* = 2.97, *P* < 0.05). In the living patients in the R337H+ group, a significantly lower mean age at diagnosis was observed compared to the R337H− group (47.90 ± 9.92 and 57.94 ± 15.9 years, respectively; Student’s *t*-test, *t* = 2.22, *P* < 0.05). No other clinical-histopathological parameters were significantly associated with the R337H mutation status (Table 1). The analysis (multiple logistic regression) considering the R337H status and the clinical variables (age at diagnosis, tumor size, lymph node metastasis, ER, PR and HER2 receptor status), also did not show any significance. Comparison of the survival curves of 14/19 R337H+ patients and 44/50 R337H− patients indicated no significant differences between the two groups of patients (Kaplan–Meier test, *P* > 0.05).

**Table 1:** Association of the clinical and pathological variables and survival with *TP53* R337H.

Variable	*TP53* R337H+	*TP53* R337H−	*P* value
**Age (yrs)**	47.88 ± 11.56 (n = 19)	58.52 ± 15.18 (n = 50)	***t = 2.97; P*** < **0.05**
**Tumor size (cm)**	2.76 ± 1,43 (n = 19)	3.02 ± 1.62 (n = 50)	*t* = 0.62; *P* > 0.50
T1	26.31% (n = 5)	30% (n = 15)	
T2	63.15% (n = 12)	64% (n = 32)	
T3	10.5% (n = 2)	6% (n = 3)	
**Tumor grade**			
I	5.26% (n = 1)	18% (n = 9)	χ^2^_2_ = 2.074; *P* > 0.30
II	42.10% (n = 8)	50% (n = 25)	
III	42.10% (n = 8)	32% (n = 16)	
**Tumor stage**			
I	15.8% (n = 3)	15.9% (n = 7)	χ^2^_2_ = 0.70; *P* > 0.80
II-A	15.8% (n = 3)	29.5% (n = 13)	
II-B	26.31% (n = 5)	36.36% (n = 16)	
III-A	10.52% (n = 2)	9.1% (n = 4)	
**Lymph node metastasis**			
Positive	42.1% (n = 8)	56% (n = 28)	χ^2^_1_ = 0.03; *P* > 0.10
Negative	36.8% (n = 7)	44% (n = 22)	
**ER expression**			
Positive	52.63% (n = 10)	80% (n = 40)	χ^2^_1_ = 1.15; *P* > 0.20
Negative	26.3% (n = 5)	20% (n = 10)	
**PR expression**			
Positive	47.36% (n = 9)	84% (n = 42)	χ^2^_1_ = 2.62; *P* > 0.10
Negative	26.31% (n = 5)	16% (n = 8)	
**HER2 over expression**			
Positive	21.05% (n = 4)	28% (n = 14)	χ^2^_1_ = 0.03; *P* > 0.80
Negative	47.36% (n = 9)	72% (n = 36)	
**Patients alive**	63.2% (n = 12)	64% (n = 32)	
Age at diagnosis	47.90 ± 9.92	57.94 ± 15.82	***t = 2.22; P*** < **0.05**
Survival (mos)	59.25 ± 46.14	69.74 ± 42.47	*t* = 0.72; *P* > 0.40
**Patients deceased**	17% (n = 2)	21% (n = 9)	
Age at diagnosis	55 ± 9.89	56.78 ± 11.21	
Survival (mos)	16 ± 11.31	37.22 ± 23.39	*t* = 1.21; *P* > 0.20

### Analysis of copy number alterations (CNAs)

To determine the patterns of CNAs that could be influenced by the *TP53* R337H variant, we performed genome-wide array-comparative genomic hybridization (CGH) analysis using an oligonucleotide array-CGH platform (Agilent Technologies, Inc.). This analysis was conducted in nine R337H+ of the 19 breast cancer patients and in nine R337H− patients.

In the R337H+ cases, 467 CNAs were observed, with an average of 51.89 ± 33.29 alterations per case. The gains of copy number were more frequent, accounting for 57.6% (269/467) of all alterations. The main cytobands with CNAs (> 30% of cases) observed in this group were 8q11.1–q24.3 (89% of cases), 8p12–p11.21 (78%), and 1q21.1–q44 (67%), followed by 1p36.33–p36.32, 6p25.3–p21.1, 8q24.3, 11p15.5, 11q13.4–q25, 13q11–q34, 14q21.1–q32.33, 14q32.33, 17p13.3–p11.2, 19p13.3, 19p13.11, and 20q11.21 (44%). Among these cytobands (except at 11q, 13q, and 14q21.1–q32.33, which were observed with loss of copy number), 8p12–p11.21 and 19p13.3 were observed with high levels of gain (log 2 > 2.0). The cytobands 7p22.3, 9q34.11, 21q22.3, 22q11.1–q13.3, 22q11.22, and 22q13.31 were also observed with high levels of gains and were present in > 30% of the cases.

In the R337H− group, 204 CNAs were observed, with an average of 22.67 ± 16.78 alterations per case. The gains of copy number were more frequent, accounting for 82.75% (128/204) of all alterations. The main cytobands with CNAs observed in this group were 14q32.33 (89% of cases), 8p11.22, 8q11.1–q24.3 and 22q11.22 (78%), 7p22.3 (67%), 1p36.33–p36.32 (56%), and 8q24.3, 10q26.3, 16p13.3–p11.1, and 17p13.3–p11.2. In these cytobands (except 17p, which was observed with loss of copy number), high levels of gains (log 2 > 2.0) were observed in 8p11.22 and 22q11.22.

Finally, the comparison of the total number of CNAs in both groups, which was measured by the comparison of the total “number of calls” in the Cytogenomics aberration interval base reports, revealed a significantly higher number of CNAs in the R337H+ group of patients (*t* = 2.35; *P* < 0.05). The findings indicated that genomic instability was more frequent in patients with the R337H variant. The main cytobands with CNAs (> 30% of the cases) observed in each group of patients are presented in Table 2.

**Table 2:** Main affected cytobands (> 30% of the cases) and their corresponding patterns of CNAs in both groups of patients analyzed.

Cytoband	R337H+		R337H−		Cytoband	R337H+		R337H−	
	CNAs	n (%) cases	CNAs	n (%) cases		CNAs	n (%) cases	CNAs	n (%) cases
1p36.13	–	0	Gain	3 (33)	12q12–q24.33	Gain	3 (33)	–	0
1p36.33–p36.32	High gain	4 (44)	Gain	5 (56)	13q11–q34	Loss	4 (44)	–	0
1q21.2–q44	Gain	6 (67)	–	0	13q34	High gain	3 (33)	–	0
2q11.1–q37.3	Gain	3 (33)	–	0	14q21.1–q32.33	Loss	4 (44)	–	0
4p16.1	Gain	3 (33)	Gain	3 (33)	14q32.33	Gain	4 (44)	Gain	8 (89)
4q22.2–q28.1	Gain	3 (33)	–	0	16p13.3–p11.2	Gain	3 (33)	Gain	3 (33)
6p22.1–p11.2	Loss	3 (33)	–	0	16q11.2–q24.3	Loss	3 (33)	–	0
6p25.3–p21.1	Gain	4 (44)	–	0	16q24.2–q24.3	Gain	3 (33)	–	0
6q11.1–q27	Loss	3 (33)	–	0	17p13.3–p11.2	Loss	4 (44)	Loss	4 (44)
7p15.3–p12.3	Gain	3 (33)	–	0	17q11.1–q25.3	Gain	3 (33)	–	0
7p22.2–p15.3	Gain	3 (33)	–	0	17q25.3	Gain	3 (33)	Gain	3 (33)
7p22.3	High gain	3 (33)	Gain	6 (67)	18q11.1–q12.3	Gain	3 (33)	–	0
7q11.21–q36.3	Gain	3 (33)	–	0	19p13.11	High gain	4 (44)	–	0
8p11.22	–	0	High gain	7 (78)	19p13.3	Loss	4 (44)	–	0
8p12–p11.21	High gain	7 (78)	–	0	19p13.3	Gain	3 (33)	–	0
8p23.3–p12	Loss	3 (33)	Loss	3 (33)	19q13.2–q13.33	Loss	3 (33)	–	0
8q11.1–q24.3	Gain	8 (89)	Gain	7 (78)	19q13.33–q13.43	Gain	3 (33)	–	0
8q24.3	Gain	4 (44)	Gain	4 (44)	20q11.21	Gain	4 (44)	–	0
9p24.3–p13.1	Loss	3 (33)	–	0	20q13.12–q13.33	Gain	3 (33)	Gain	3 (33)
9q34.11	High gain	3 (33)	–	0	20q13.33	Loss	3 (33)	–	0
9q34.2–q34.3	Gain	3 (33)	Gain	3 (33)	20q13.33	Gain	3 (33)	–	0
10q21.3–q26.3	Loss	3 (33)	–	0	21q22.3	High gain	3 (33)	Gain	3 (33)
10q26.3	Gain	3 (33)	Gain	4 (44)	22q11.1–q13.33	High gain	3 (33)	–	0
11p15.5	Gain	4 (44)	Gain	3 (33)	22q11.21	Gain	3 (33)	Gain	3 (33)
11q12.2–q14.1	Gain	3 (33)	–	0	22q11.22	High gain	3 (33)	High gain	7 (78)
11q13.3–q13.4	–	0	Gain	3 (33)	22q13.31	High gain	3 (33)	–	0
11q13.4–q25	Loss	4 (44)	–	0	Xq28	Gain	3 (33)	–	0

### Functional enrichment pathways

To determine the function of the microRNAs (miRNAs) mapped in these cytobands (Table S1) that could be affected by the presence of CNAs, pathway enrichment analysis was performed using DIANA-miRPath v.3.0^27^. MiRNAs corresponding to target genes and the main signaling pathways involved were identified. Due to the large number of cytobands affected, especially in the R337H+ group of patients, only cytobands affected more than 50% of the cases were considered.

In the R337H+ group of patients, 76 significant Kyoto Encyclopedia of Genes and Genomes (KEGG) pathways were observed (*P* < 0.05). Among the top 15 pathways observed (based on P-value), those affected by the largest number of miRNAs were the “Pathways in cancer” and “Proteoglycans in cancer,” with 82 miRNAs and 79 miRNAs, respectively. In this group, the p53 signaling pathway was among the significant pathways involved, being affected by 55 miRNAs (Table 3).

**Table 3:** Top 15 pathways, their corresponding number of genes and miRNAs, observed to be affected by the main CNAs, in the two groups of breast cancer patients analyzed (presented by the number of affected miRNAs).

R337H+ breast cancer patient			
KEGG pathway	#MiRNAs	#Genes	*P* value
Pathways in cancer	82	318	8.80E−04
Proteoglycans in cancer	79	171	1.24E−02
Hippo signaling pathway	77	125	1.57E−06
Focal adhesion	76	175	5.28E−05
Signaling pathways regulating pluripotency of stem cells	76	122	6.29E−03
Oxytocin signaling pathway	72	132	6.95E−06
Axon guidance	72	111	6.29E−03
TGF-beta signaling pathway	67	70	5.28E−05
ErbB signaling pathway	66	79	2.55E−05
Renal cell carcinoma	65	62	2.47E−04
Pancreatic cancer	62	59	4.95E−06
Glioma	62	57	1.57E−06
ECM-receptor interaction	62	70	6.29E−03
Colorectal cancer	60	57	4.51E−06
Long-term depression	60	51	2.08E−03
p53 signaling pathway	55	60	2.56E−02

R337H− breast cancer patient			
KEGG pathway	#MiRNAs	#Genes	*P* value
Proteoglycans in cancer	10	65	1.74E−03
Hippo signaling pathway	10	44	4.30E−03
Ras signaling pathway	10	69	6.19E−03
Signaling pathways regulating pluripotency of stem cells	10	40	5.23E−02
Pathways in cancer	10	97	8.69E−02
Axon guidance	10	38	1.84E−01
Focal adhesion	10	59	1.84E−01
Thyroid hormone signaling pathway	10	31	1.84E−01
ErbB signaling pathway	9	34	6.29E−06
Regulation of actin cytoskeleton	9	64	5.23E−02
Amphetamine addiction	9	21	4.30E−03
Glioma	8	26	5.13E−06
Lysine degradation	8	16	6.29E−06
Renal cell carcinoma	7	24	7.33E−02
Synaptic vesicle cycle	6	18	2.27E−01

KEGG, Kyoto Encyclopedia of Genes and Genomes; ECM, Extracellular Matrix.

In the R337H− group of patients, 26 significant pathways were observed (*P* < 0.05). Among the top 15 pathways observed (based on *P* value), the ones affected by the largest number of miRNAs (10 miRNAs) were “Proteoglycans in Cancer, Hippo, Ras, Pluripotency stem cells regulating and Thyroid hormone signaling pathways, Pathways in cancer, Axon guidance and Focal adhesion” (Table 3).

**Figure 1:**
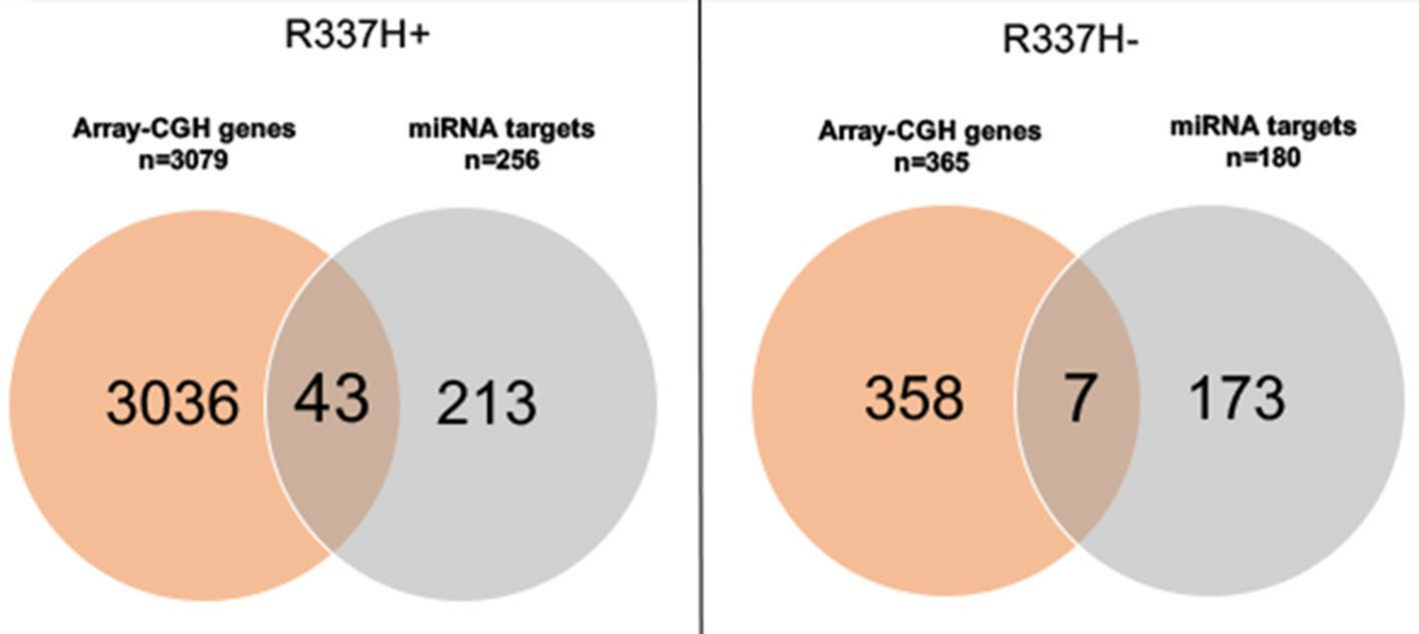
Venn diagram showing the integration of genes located at the cytobands most affected by CNAs and the target genes regulated by the miRNAs mapped at the cytobands in the *TP53* R337H+ (left) and R337H− (right) group of patients.

Finally, we identified the miRNA targets predicted to be regulated by these miRNAs, using Tarbase v.7.0, miRNA target gene (miTG) scores > 0.7 indicated microT-CDS interactions^28^. Only genes correlated with miRNAs that presented strong evidence in the validation methods cited in the Materials and Methods section were considered. This analysis revealed 256 and 180 miRNA targets for the R337H+ and R337H− groups, respectively. Upon integration with the genes that were identified in the affected cytobands by array-CGH (3079 in the R337H+ group and 365 in the R337H− group), we observed that there were 43 genes in common in the R337H+ group and seven genes in the R337H− group (Fig. 1, Table S2). Four genes (*CCNE2*, *MTDH*, *RDH10,* and *SNAI2*) were commonly observed in both the R337H+ and R337H− groups of patients. The genes affected in the R337H+ group were in the chromosome regions. These genes were mainly affected by CNAs in these cases as revealed by the array-CGH analysis, including 1q21–q44, 2q11-37.3, 8q21–q24, 16q23.2, and 17q25.3. Interestingly, in the R337H− group, all the genes identified in the integration analysis mapped at the 8q region, and were commonly affected in these cases in the array-CGH analysis. These results indicated that CNAs could affect genes that are also potentially regulated by miRNAs.

### Association of the target miRNA genes with survival using KMPlot database

The genes identified in both groups of patients in the aforementioned integrative analysis were queried in the KM Plot database to determine their potential association with survival outcome in breast cancer patients. This analysis was performed by querying the database in all the groups of breast cancer cases available and in breast cancer cases based on the *TP53* variants status. The type of *TP53* variants in the cases was not disclosed (Table S2).

In the R337H+ group, 72.1% (31/43) of the genes were associated with survival. Seventeen and 14 of the genes were identified in patients with higher and lower survival rates, respectively. Five genes in this group were only observed in cases that presented with *TP53* variants in the KMplot database. Overexpression of three of these genes (*ECM1, MMP16,* and *CTHRC*) was associated with significantly worse survival. Overexpression of *MCL1* and *STAT1* was associated with better survival***. ***Four other genes (*ITGA6*, *HOXD10*, *FASN,* and *BUP1*) were observed only in cases that were negative for *TP53* variants in the KMplot database. Higher expression of *ITGA6* was significantly associated with better survival. Higher expression of *HOXD10*, *FASN,* and *BUP1* was associated with worse survival.

In the R337H− group of patients, 85.7% (6/7) genes were associated with survival. Only *SNAI2* (also found in the R337H+ group*)* was not associated with survival. Overexpression of *OXR1* was the only gene in this group that was significantly associated with *TP53* status, conferring a higher survival rate in patients with no *TP53* variants. Interestingly, in the R337H+ group, three genes (*IGFBP5, MAF,* and *SMYD3*) that were not associated with survival in the breast cancer cases in general were associated with survival specifically in cases with *TP53* variants.

### Copy number variations (CNV) analysis

To further verify the differences in the level of genomic instability in R337H+ cells, we performed genome-wide CNVs analyses in homozygous (R337H+/R337H+) and heterozygous (Wt/R337H+) fibroblast cell cultures. Specific targets (9q, 9q33–34, 11p, and 11p15) considered to harbor genes potentially involved in adrenocortical (ACT)^29,30^ and breast cancer^31,32^ tumorigenesis were evaluated (Table 4). Statistically significantly increased levels of CNVs (all *P* < 0.05) in chromosomes 1, 2, 3, 6, 7, 8, 10, 11, 13, 14, 15, 16, and 22 were found only in homozygous R337H/R337H cells when compared to the other cells. Remarkably, the most consistent losses and gains were identified in 11p, suggesting that this is a susceptible target to CNV, which is also known to be related to IGF-2 overexpression in ACTs.

**Table 4:** Comparisons* of the CNV means between homozygous (R337H+/R337H+) and heterozygous (Wt/R337H+) human fibroblasts.

CNV > 10 kb					CNV > 50 kb					CNV > 100 kb				
Chr	G/L	Hetero	Homo	*P* value	Chr	G/L	Hetero	Homo	*P* value	Chr	G/L	Hetero	Homo	*P* value
**All chromosomes**														
chr1	G + L	4.7	20.4	0.022	chr1	L	0.5	1.5	0.038	chr10	L	0	0.7	0.046
chr1	G	10.1	42.0	0.033	chr1	G + L	2.5	6.7	0.033	chr14	G + L	0.8	1.8	0.028
chr2	G	4.7	14.7	0.027	chr2	G + L	2.7	5.8	0.049	chr15	L	0	0.7	0.046
chr3	G	4.2	16.8	0.043	chr3	G	0.8	3.4	0.006	chr15	G + L	1.7	3.8	0.017
chr6	G	2.3	7.0	0.040	chr3	G + L	1.8	6.1	0.037	chr15	G	0.5	2.3	0.016
chr7	G	1.2	25.3	0.032	chr5	G	0.3	2.0	0.028	chr16	G + L	0.5	2.4	0.016
chr8	G	3.0	15.7	0.041	chr7	G	0.3	4.7	0.028					
chr10	G	6.3	32.0	0.030	chr7	G + L	0.5	6.1	0.004					
chr10	G + L	8.0	37.8	0.022	chr10	G + L	1.8	6.4	0.038					
chr11	G	2.7	13.3	0.042	chr14	G + L	1.8	5.7	0.031					
chr11	G + L	4.8	24.1	0.034	chr15	G	2.1	4.7	0.023					
chr13	G	0	2.6	0.014	chr15	L	0.7	3.1	0.043					
chr14	G	2.5	6.1	0.029	chr15	G + L	2.8	7.8	0.008					
chr15	G	3.5	8.4	0.041	chr16	G	1.0	4.8	0.024					
chr16	G	2.6	18.8	0.040	chr16	G + L	1.3	6.4	0.048					
chr22	G + L	2.3	19.1	0.029	chr19	L	0	1.7	0.045					
					chr20	G + L	0.2	1.0	0.041					
**Specific targets (11 and 11p15)**														
chr11p	G	1.3	6.3	0.039	chr11p	G + L	0.3	2.4	0.048	chr11p	L	0	0.7	0.046
chr11p	L	0.06	4.7	0.032	chr11p15 G + L		0	1.3	0.048					
chr11p	G + L	2.0	11.0	0.014										
chr11p15 G		0.5	5.0	0.023										

Only statistically significant differences (*p* < 0.05) are shown.

*The comparisons were performed between homozygous (n = 8, 4 treated and 4 untreated assays) and heterozygous (n = 8, 4 treated and 4 untreated assays). Chr, chromosomes; G, gain; L, loss.

## Discussion

In this study, we report the frequency of the *TP53* R337H variant in tumor tissues of patients with no family history of breast cancer, and its association with clinical and histopathologic parameters and survival outcome. We also conducted a comprehensive computational analysis to determine the impact of the *TP53 R337H* variant on genomic instability, evaluating both the breast tumor tissue of the patients and homozygous (R337H+/R337H+) and heterozygous (Wt/R337H+) fibroblast cell cultures established from a patient with adrenocortical cancer and a variant carrier, respectively.

The frequency of the *TP53* R337H variant was observed in 2.36% of the sporadic breast cancer cases we evaluated, with an increase of 7.71 in genotypic frequency (2.36% versus 0.306%) when considering all 214,087 newborns tested by Custódio et al.^5^ and Costa et al.^4^. This is the first report on the R337H genotypic frequency in breast cancer tissue without family history of cancer in Paraná state. Previous studies have evaluated the frequency of the R337H variant in women with and without breast cancer in different regions of Brazil. Palmero et al.^22^ evaluated 750 healthy women from Porto Alegre in southern Brazil and identified a genotypic frequency of 0.30%. Gomes et al.^14^ reported an R337H genotypic frequency of 0.5% (2/390) in women diagnosed with breast cancer in Rio de Janeiro in, southeast Brazil. This frequency was expected, considering that Rio de Janeiro is more distant from the clustered R337H region formed by four states (Rio Grande do Sul, Santa Catarina, Paraná, and São Paulo). Giacomazzi et al.^12^ reported a higher genotypic frequency (8.6%, 70/815) in a cohort of breast cancer patients from Rio Grande do Sul and São Paulo without family cancer history. These cases were from referral cancer centers in Porto Alegre and São Paulo, which could account for the higher frequency. Other possibilities are associations with other genetic variants that facilitate carcinogenesis among R337H carriers or their exposure to environmental factors in the regions where they live, as demonstrated for ACTs^4^. Interestingly, the frequency of the breast cancer patients with the R337H variant in our study varied according to the hospitals and regions, ranging from 1.57% in Curitiba, the capital of Paraná, to 7.6% in the eastern Paraná state, where agricultural activities are more prominent. It is important to point out, however, that in this study only the *TP53* R337H variant was genotyped. Therefore, it is very likely that both the R337H carriers and non-carriers groups, harbor other somatic and/or *TP53* germline variants.

The association of clinical parameters in the groups of breast cancer patients we studied based on the R337H variant revealed a lower average age (47.88 ± 11.56) at diagnosis of the R337H+ group compared to the R337H− group (47.88 ± 11.56 vs. 58.52 ± 15.18 years; *P* < 0.05). Younger age at diagnosis was also observed by Giacomazzi et al.^12^ and Andrade et al.^21^ in patients with breast cancer positive for the R337H variant. A previous study reported that 12.1% of the patients diagnosed with breast cancer were diagnosed before 45 years of age compared to 5.1% diagnosed after 55 years of age. The same was observed in the study by Gomes et al.^14^, in which two cases of breast cancer with the R337H variant had a lower age at diagnosis. In these cases, however, both patients had a family history of breast and/or other types of cancer. Altogether, the present and prior studies provide evidence of an association between the R337H variant and early age of diagnosis, regardless of a family history of cancer.

To determine the impact of the R337H variant on the tumor genome stability of breast cancer patients, we performed a genome-wide evaluation of CNAs. Genomic instability is a hallmark of cancer hallmark that can be evident as the presence of chromosome regions with copy number gains/amplifications and losses/deletions^25,26^. These alterations can directly affect the expression of genes and miRNAs mapped at these chromosome regions^33,34^. In particular, miRNAs have been shown to be commonly affected targets for genomic instability^35,36^, which can significantly modulate tumor progression, through the regulation of critical cancer genes, such as the *TP53*^37^.

In our analysis, we observed a significantly higher frequency of CNAs in the R337H+ breast cancer patients than in the R337H−  group. In addition, a significantly higher number of cases in the R337H+ group displayed the main affected cytobands compared to the R337H− group. These results showed a higher level of genomic instability in the R337H+ patients and a preferential involvement of the most affected cytobands. Not surprisingly, as demonstrated by KEGG pathway enrichment analysis based on the mapping of genes and miRNAs in these affected cytobands, we observed functional signaling pathways that were potentially affected in both groups of patients. However, a larger number of these pathways were observed in the R337H+ group. Critical cancer-associated pathways among the top 15 most significantly affected, such as Proteoglycans in cancer, Pathways in cancer, ErbB, Hippo, and Ras pathways, were observed in both groups, likely not reflecting the presence of the R337H variant but the tumorigenic process itself.

Interestingly, in the R337H+ group, the *TP53* and *TGFB* signaling pathways, which were not observed in the R337H− group, were among the pathways mostly affected by the miRNAs present in the cytobands with CNAs. Crosstalk between these pathways, via the Smad signal transduction pathway^38^, has been reported, and although the mechanisms involved remain to be fully elucidated, additional signaling pathways, such as phosphoinositide 3-kinase/AKT and extracellular signal-regulated kinase^39,40^ and the involvement of miRNA regulation^41^ have been suggested.

As cited above, considering that CNAs are one of the mechanisms that affect miRNA expression (and thus miRNA target expression)^33,34^, we next identified genes that were potentially altered by CNAs and that were also targets of the miRNAs mapped in these main affected regions. In the R337H+ and R337H− groups, we observed 43 and seven genes (four genes were common to both groups), respectively, which could be affected by these two distinct molecular mechanisms. The genes affected in the R337H+ group were located in the cytobands, which were mainly affected by CNAs in these cases, such as 1q21.2, 1q44, 2q13, 2q31.1, 2q32.2, 2q35, 8q21.3, 8q22.3, 8q23.1, 16q23.2, and 17q25.3 (in the R337H− group, only genes mapped at 8q were observed in this integration analysis). Although the impact of such alterations in gene and miRNA expression has to be confirmed in experimental expression assays, the observations support the finding that CNAs can affect genes that are also potentially regulated by miRNAs^33,34,41–44^. Several of these genes were previously identified as members of the main signaling pathways observed and, interestingly, displayed direct protein interactions with p53 (data not shown—String Network v. 11.0 (https://string-db.org/). These genes included *BUB1*, *CCNE2*, *MCL1*, *MYC*, *SNAI2,* and *STAT1*. Several miRNAs mapped at the above cytobands have been previously reported as regulators of *TP53* gene expression in tissue samples of patients with sporadic breast cancer^45^. However, little is known about the roles of miRNAs in patients with *TP53* variants. A limited number of reports in cancer have shown the phenotypic consequences of variant’s *TP53* upon miRNA binding^46–49^, such as gain of function of the R157H and miR-128^46^, and R273H and miR-27a^48^. In our study, in the R337H+ group the miR-128 was among the miRNAs previously described in these studies^46^. However, no miRNA present in the R337H− group was previously associated with *TP53* variants. One recent study has shown the association of a polymorphism in miR-605 with the occurrence of multiple primary tumours in R337H carriers that meet the LF criteria^50^. However, this miRNA, which mapped at the 10q21.1 cytoband, was not among the main regions affected by CNAs in any of the groups of the patients in this study.

The query of the KMplot database of the genes we identified after the integration analysis above could indicate their association with survival of breast cancer patients. The analysis in the R337H+ group revealed significant associations with 72.1% of the genes, five of which (*CTHRC1, ECM1, MCL1, MMP16*, and *STAT1*) were associated specifically in cases that presented with *TP53* variants in the KMplot database. Four other genes (*ITGA6*, *HOXD10*, *FASN,* and *BUP1*) were observed only in the breast cancer cases in the database that were negative for *TP53* variants. In addition, three genes (*IGFBP5, MAF,* and *SMYD3*) that were not associated with survival in breast cancer cases in general were associated with survival specifically in cases with *TP53* variants*.* In the R337H− group of patients, only *OXR1* was significantly associated with *TP53* status. Interestingly, this gene (Human Oxidation Resistance 1), originally identified as a protein that decreases genomic mutations in *Escherichia coli*^51^, prevented reactive oxidation species formation and reduced the duration of gamma-ray-induced G2/M arrest in HeLa cells. Altogether, these data indicated that *OXR1* prevents genome instability and could function in the cases with R337H−. It is important to point out, however, the limitation of the interpretation of the KMplot results in relation to the R337H variant impact on survival of breast cancer patients, considering that there was no description in the queried database of the type of *TP53* variants in breast cancer cases.

Finally, to further verify the impact of the R337H variant on the level of genomic instability, CNVs were analyzed in homozygous (R337H+/R337H+) and heterozygous (Wt/R337H+) normal fibroblasts exposed to a DNA damage agent. These analyses revealed a significant increase in CNVs in the cells homozygous for the R337H variant compared to cells with one wild-type *TP53* allele. Although these variations were significant in several chromosomes, reflecting a genome-wide instability, our data revealed that chromosome 11p was the region most susceptible to CNV (> 10 kb, > 50 kb, and > 100 kb), which is consistent with the CNAs described in ACTs^29,30^ and breast cancer^31,32^. At 11p15, in particular, a large cluster of imprinted genes that includes *IGF2, a* paternally expressed fetal growth factor, can be altered in ACTs, including those with the R337H variant^52^. Previous studies have shown the additional impact of CNVs on tumors harboring germline *TP53* variants, such as R337H^53–56^. Letouzé et al.^56^ analyzed 25 ACT tumors, 13 of which with the R337H variant. The authors utilized high-resolution single nucleotide polymorphism analysis to demonstrate that the cases with the wild-type *TP53* displayed distinct genomic profiles, with significantly fewer rearrangements, compared to the cases with the R337H variant. This finding was also observed in patients with Li-Fraumeni, where an increased number of CNVs were observed in patients carrying germline variants in the *TP53* gene, such as R337H^55^.

In conclusion, the *TP53* R337H variant may contribute 2.36% of all breast cancer cases without family cancer history in Paraná state of Brazil. Among other mechanisms, R337H increases the level of breast cancer genomic instability, as evidenced by the presence of a higher number of CNAs potentially affecting genes/miRNAs that regulate critical cancer signaling pathways. This instability was also observed in R337H+/R337H+ fibroblast cells, which showed a significant increase in CNVs compared to cells with one wild-type *TP53* allele. Altogether, these results indicate that the presence of the R337H variant is associated with an increased level of genomic instability in the cells. However, its direct role in modulating breast cancer tumorigenicity is unknown.

## Materials and methods

### Sample collection

A total of 805 breast tissue samples from different patients, predominantly of European descent, who had been diagnosed with breast cancer were collected during primary surgery at Hospital Nossa Senhora Das Graças (HNSG) and Hospital de Clínicas (HC), both from Curitiba and União Oeste Paranaense de Estudos e Combate ao Câncer (UOPECCAN), Cascavel, southern Brazil. All patients provided signed informed consent. Among these samples, 418 and 105 were of fresh tissue acquired from the Human Cytogenetics and Oncogenetics Laboratory Biorepository (collected at HNSG and HC), and the UOPECCAN Biorepository, respectively, and 282 were from paraffin-embedded formalin-fixed (FFPE) tissue blocks acquired from the HNSG Pathology Service.

Clinical and histopathologic data of the patients were collected directly from the medical records in a coded manner without patient identifiers. The majority of the patients (n = 584, 74.5%) were diagnosed with invasive ductal carcinoma, followed by invasive lobular carcinoma (8.1%) and in situ ductal carcinoma (7.5%). Other types of carcinomas were present in 8.4% of the cases. Other clinical and histopathologic data collected included age at diagnosis, tumor size, stage and grade, estrogen, progesterone, and HER2 receptor status, and presence of lymph node metastasis. Survival data (alive or deceased) were obtained for 58 patients. Survival time was evaluated in months from the date of diagnosis until the last medical visit. These parameters were evaluated and compared for the patients studied according to the *TP53* R337H variant status (Table 1).

### *TP53* R337H variant genotyping

Genotyping for the *TP53* R337H variant (NM_000546.6(TP53):c.1010G > A(p.Arg337His—National Center for Biotechnology Information. ClinVar; [VCV000012379.9] was performed for all 805 patients by TaqMan Real-Time PCR. DNA from fresh tumor tissue was isolated using the phenol–chloroform method as per standard protocols. For FFPE samples, DNA isolation was performed using the protocol previously optimized by our group^57^.

Genotyping was performed using TaqMan hydrolysis probes. Two probes annealing to the codon 337 of the *TP53* gene were designed. One corresponded to the normal allele and the other to the mutated allele. Reactions were prepared in 96-well plates, which contained three controls (two “blanks” and one individual homozygous for the R337H variant). All reactions contained three controls (two “blanks” and one individual homozygous for R337H variant). Each reaction contained 5 µL Master Mix Universal (2 ×) + 2.5 µL ultrapure water + 0.5 µL customized probe (10×) + 60 ng DNA (3 µL, 20 ng/µL). PCR was conducted in a ViiA 7 Real-Time PCR apparatus (Applied Biosystems, Foster City, CA, USA) under the following cycling conditions: 95 °C for 10 min, 35 cycles at 95 °C for 15 s, 60 °C for 30 s, and 72 °C for 1 min.

Sanger sequencing was used to confirm the positive results from genotyping, and it was performed using BigDye Terminator v3.1 Cycle Sequencing Kit standard protocols (ThermoFisher, Waltham, MA, USA). Briefly, primers (*forward 5*′ *CCATCTTTTAACTCAGGTACTGT 3*′*/reverse: 5*′ *TGAATGAGGCCTTGGAACTC 3*′) flanking the target region were designed based on *Ensembl gene sequence* and synthesized by Integrated DNA Technologies (IDT) (Coralville, IA, USA). PCR products were directly sequenced on an ABI-3530 automated sequencer (Applied Biosystems, Foster City, CA, USA), and the software ABI-3530 Collection and Sequencing Analysis were used to perform the analysis.

### *TP53* R337H status and clinical-histopathological parameters, and survival

The association of the *TP53* R337H variant and the clinical-histopathological parameters was performed between the R337H+ and R337H− groups of patients, considering tumor grade and size, lymph node metastasis, and estrogen, progesterone, and HER2 receptor status. From the R337H− group, 50 patients were selected for this analysis following two main criteria. The first a diagnosis of invasive ductal carcinoma (the same diagnosis as the carrier group). The second was the highest amount of clinical information for the clinical and histopathological parameters above. Student’s *t*-test was performed to compare the patient groups’ age and tumor size. The chi-square test was used to compare tumor grade and stage, expression of estrogen, progesterone, and HER2 receptors, and lymph node metastasis. Multiple logistic regression analysis was performed using the software GraphPad Prim 8 and taking into consideration the clinical parameters (age at diagnosis, tumor size, lymph node metastasis, and ER, PR, and HER2 receptors as independent variables (X) and the patient R337H genotype (positive or negative for the R337H variant) as the single dependent (Y) variable. Survival data were analyzed using Student’s *t* and Kaplan Meier tests. Statistical significance was considered at *P* < 0.05.

### Genome-wide CNA analysis

To detect CNAs, as a measurement of genomic instability, the DNA from the breast cancer cases positive and negative for the *TP53* R337H variant were profiled using the SurePrint G3 Human CGH Microarray (Agilent, Santa Clara, CA, USA) according to our previous protocol for FFPE samples^57^. Nine patients were evaluated from each group of patients using the same protocol. DNA isolated from peripheral blood from multiple normal individuals was used as a control (reference) DNA. Control and case samples were directly labeled using the Bioprimer a-CGH Genomic Labeling kit and hybridized to the arrays for 40 h. The arrays were scanned using the model G2565CA scanner (Agilent). The data were extracted using Feature Extraction software v10.10 (Agilent). The Agilent Cytogenomics v.5.0 software was used to analyze the data using the algorithm ADM-2, threshold of 6.0, and an aberration filter with a minimum of three probes. Copy number gains and losses were defined as the minimum average absolute log2 ratio (intensity of the Cy5 dye (reference DNA)/intensity of the Cy3 dye (test DNA) value of ≥ 0.25 and <  − 0.25, respectively. High copy number gains and losses were considered for log2 ratios ≥ 2.0 or < 2.0, respectively. The number of “calls” (total significant number of CNAs) and the specifically affected cytobands were obtained from the generated aberration interval base reports (Agilent Cytogenomics v.5.0). Only cytobands affected in > 30% of the cases were considered. Statistical analysis of the cytobands and number of calls was performed using the GraphPad Prism software v. 6.0.

### Functional enriched pathways

For both groups of breast cancer patients (*TP53* R337H+ and R337H −) analyzed by array-CGH, the identification of the genes and miRNAs mapped in the cyto-bands that were mostly affected by CNAs was obtained from the Agilent Cytogenomics v.5.0 interval base reports (based on the analysis parameters described above). DIANA-miRPath v.3.0^27^ was used to perform pathway enrichment analysis, based on the KEGG database (https://www.genome.jp/kegg). Only miRNA/mRNA targets that presented a miRNA Target Gene (miTG) score > 0.7 based on the microT-CDS^27^ interactions were included. For the selection of the main targets, only those that presented strong evidence in validation methods (luciferase assays, western blotting, and qPCR) were considered, according to miRTarBase v.7.0^28^. A direct integration of the miRNA target genes mapped in the most affected cytobands was performed, as previously described^42,44^ to determine whether the genes also mapped in these regions were miRNA targets, and therefore could be potentially affected by both CNAs and miRNA expression regulation.

### Kaplan–Meier plot analysis

The KM Plotter Tool (https://kmplot.com/analysis/) was used to calculate hazard ratios, confidence intervals, and log-rank *P* values for the selected genes resulting from the integration of the genes that were miRNA targets and also affected by copy number alterations (CNAs). This analysis was performed in relation to survival in the aggregated breast cancer clinical studies extracted from The Cancer Genome Atlas and Molecular Taxonomy of Breast Cancer International Consortium (METABRIC) databases (breast cancer cases in general and selected for *TP53* variants).

### DNA damage induction in normal fibroblasts homozygous and heterozygous for the *TP53* R337H variant

Skin biopsies from two *TP53* R337H+/R337H+ homozygous boys who had adrenocortical cancer and their heterozygous Wt/R337H+ mothers. The skin biopsies collected were from surgical removal of the foreskin for therapeutic and prophylactic reasons with the authorization by their parents. Fibroblast cultures (at 37 °C, 5% CO_2_) were obtained from the skin biopsies using Dulbecco's modified Eagle's medium (DMEM F12) supplemented with 10% fetal bovine serum and antibiotics (Sigma-Aldrich, St. Louis, MO, USA). The cultures were treated with doxorubicin twice, at passage 6 for 5 days at a concentration of 0.025 μM, and after recovering for 15 days in normal medium at a concentration of 0.0125 μM for 5 days. The cells were allowed to recover for 8 days, and DNA was isolated together with controls (untreated cells-passage 5). Each cell type, including untreated controls and doxorubicin treated (R337H+/R337H+, Wt/R337H+) were prepared in duplicate, considering that the variation of the results in most of these assays were not significant. In few cases, where differences in the cell counting were observed, the entire assay was repeated.

### Genome-wide copy number variation (CNV) analysis

Genomic DNA was isolated from all the samples (n = 4 for each cell type, with two doxorubicin treated and two untreated samples) for CNV analysis, using the Affymetrix 6.0 array. The data were analyzed using the Affymetrix Genotyping Console software in the Affymetrix Power Tool (https://www.affymetrix.com/partners_programs/programs/developer/tools/powertools.affx), using the following criteria: < 90% genotype call rate or minor allele frequency < 5% or Hardy–Weinberg equilibrium exact *P* value < 0.05 in cases or controls. The CNVs were estimated using two software programs: APT and PENNCNV^58,59^. CNV analyses were performed for each of the chromosomes, or for specific cytobands (9q, 9q33–34, 11p, 11p15, 17p and 17p13) considering their relevance to the ACTs^29,30^ and breast cancer^31,32^, as we previously described. CNVs identified in cases with > 10% overlap with CNVs identified in the controls were not considered. The CNVs identified were checked in the Database of Genomic Variants (https://projects.tcag.ca/variation). The comparison of the CNV mean values among the treated and non-treated cases was performed using the two-tailed Student’s *t-test*. A *P*-value < 0.05 was considered significant.

## Supplementary information

Supplementary tables.
