# Supplementary material for: Frequency of the TP53 R337H variant in sporadic breast cancer and its impact on genomic instability
Source: Sci Rep. 2020 Oct 6;10:16614. doi: 10.1038/s41598-020-73282-y (PMC7539008; doi:10.1038/s41598-020-73282-y)
Supplement: Supplementary file 1 — Supplementary tables. [file 41598_2020_73282_MOESM1_ESM.pdf]

Frequency of the *TP53* R337H mutation in sporadic breast cancer and its impact in genomic instability

Carolina Mathias[1], Stefanne Bortoletto[2], Ariana Centa[2], Heloisa Komechen[2], Rubens S. Lima[3], Aline S. Fonseca[2], Ana Paula Sebastião[4,5], Cícero A. Urban[3], Emerson W. S. Soares[6], Carolina Prando[2], Bonald C. Figueiredo[2], Iglenir J. Cavalli[1], Luciane R. Cavalli[2,7*] and Enilze M.F.S. Ribeiro[1]

[1]Departamento de Genética , Universidade Federal do Paraná, Curitiba, Paraná, 81531980, Brazil
[2] Instituto de Pesquisa Pelé Pequeno Príncipe, Faculdades Pequeno Príncipe, Curitiba, Paraná, 80250060, Brazil
[3] Centro de Doença da Mama, Hospital Nossa Senhora das Graças, Curitiba, Paraná, 80810040, Brazil
[4] Departamento de Patologia, Hospital de Clínicas, Universidade Federal do Paraná, Curitiba, Paraná, 81531980, Brazil
[5] Serviço de Patologia, Hospital Nossa Senhora das Graças, Curitiba, Paraná, 80810040, Brazil
[6] União Oeste Paranaense de Estudos e Combate ao Câncer, Cascavel, Paraná, 85806300, Brazil
[7] Lombardi Comprehensive Cancer Center, Georgetown University, Washington, DC, 20007, USA

* To whom correspondence should be addressed.
Tel: +5541331035; Email: luciane.cavalli@pelepequenoprinicpe.org.br; lrc@georgetown.edu

Table S1. MicroRNAs located at each cytoband affected by CNAs (> 30% of the cases) in the *TP53* R337H+ and *TP53* R337H- breast cancer groups (as per Cytogenomics v.5.0 software (Agilent Technologies Inc., Santa Clara, CA).

Groups of patients	Cytoband	Start	Stop	#miRNAs	miRNA
R337H-	1p36.13	16.397.917	19.011.244	0	
R337H+, R337H-	1p36.33 - p36.32*	759.762	2.938.033	3	MIR200B, MIR200A, MIR429
R337H+	1q21.2 - q44*	144.988.715	249.212.668	38	MIR4258, MIR3124, MIR3123, MIR3916, MIR1537, MIR1182, MIR3620, MIR320B2, MIR215, MIR194-1, MIR664, MIR29C, MIR29B2, MIR3122, MIR4260, MIR205, MIR135B, MIR181B1, MIR181A1, MIR1231, MIR1278, MIR548F1, MIR3121, MIR488, MIR214, MIR3120, MIR199A2, MIR3119-1, MIR3119-2, MIR1295, MIR557, MIR921, MIR3658, MIR556, MIR9-1, MIR765, MIR555, MIR92B
R337H+	2q11.1 - q37.3	95.954.278	243.041.364	52	MIR3127, MIR4265, MIR4266, MIR4267, MIR663B, MIR3679, MIR128-1, MIR933, MIR10B, MIR1246, MIR3128, MIR548N, MIR1258, MIR561, MIR1245, MIR3606, MIR3129, , MIR3130-1, MIR3130-2, MIR2355, MIR548F2, MIR26B, MIR375, MIR3131, MIR153-1, MIR3132, MIR4268 MIR1244-1, MIR1244-2, MIR1244-3, MIR1471 MIR4269, MIR149 MIR548G, MIR922, MIR570, MIR944, MIR1248, MIR1224, MIR569, MIR551B, MIR3919, MIR15B, MIR16-2, MIR548H2, MIR1280, MIR548I1, MIR198, MIR568, MIR567, MIR548A3, MIR3921
R337H+, R337H-	4p16.1	8.575.302	8.900.564	0	
R337H+	4q22.2 - q28.1	94.462.862	126.245.247	9	MIR3684, MIR367, MIR302D, MIR302A, MIR302C, MIR302B, MIR1243, MIR577, MIR1973
R337H+	6p22.1 - p11.2	27.987.770	58.614.061	8	MIR206, MIR133B, MIR586, MIR3925, MIR1275, MIR219-1, MIR1236, MIR877
R337H+	6p25.3 - p21.1	255.350	44.337.859	9	MIR3925, MIR3925, MIR1275, MIR219-1, MIR1236, MIR877, MIR3143, MIR548A1, MIR3691
R337H+	6q11.1 - q27	62.667.980	170.890.108	13	MIR3939, MIR1913, MIR3918, MIR3692, MIR3668, MIR3145, MIR3662, MIR548B, MIR2113, MIR548H3, MIR4282, MIR30C2, MIR30A
R337H+	7p15.3 - p12.3	24.038.750	48.563.988	7	MIR3943, MIR1200, MIR550A2, MIR550A1, MIR550B1, MIR196B, MIR148A
R337H+, R337H-	7p22.3*	1.643.913	1.779.622	1	MIR339
R337H+	7q11.21 - q36.3	62.618.673	158.909.738	34	MIR4283-1, MIR4283-2, MIR153-2, MIR595, MIR3907, MIR671, MIR548I4, MIR548F4, MIR548F3, MIR548T, MIR29A, MIR29B1, MIR490, MIR335, MIR182, MIR96, MIR183, MIR593, MIR129-1, MIR592, MIR3666, MIR4285, MIR25, MIR93, MIR106B, MIR591, MIR3609, MIR653, MIR489, MIR548M, MIR590, MIR4284, MIR3914-1, MIR3914-2
R337H-	8p11.22*	39.258.894	39.386.158	0	

Cont. Table S1.

Groups of patients	Cytoband	Start	Stop	#miRNAs	miRNA
R337H+	8p12 - p11.21*	36.227.477	42.697.844	2	MIR486, MIR3170
R337H+, R337H-	8p23.3 - p12	221.611	36.014.509	15	MIR4288, MIR3622A, MIR3148, MIR4287, MIR3622B, MIR320A, MIR383, MIR3926-1, MIR3926-2, MIR598, MIR1322, MIR124-1, MIR597, MIR548I3, MIR596
R337H+, R337H-	8q11.1 - q24.3*	47.681.335	146.280.020	20	MIR124-2, MIR939, MIR1234, MIR661, MIR937, MIR30B, MIR30D, MIR1204, MIR1205, MIR1206, MIR1207, MIR1208, MIR3610, MIR2053, MIR3151, MIR599, MIR875, MIR3150B, MIR3150, MIR2052
R337H+, R337H-	8q24.3	144.172.190	145.811.230	4	MIR937, MIR939, MIR1234, MIR661
R337H+	9p24.3-p13.1	500.525	39.101.408	6	MIR101-2, MIR3152, MIR491, MIR31, MIR876, MIR873
R337H+	9q34.11	131.775.305	131.830.898	0	
R337H+, R337H-	9q34.2 - q34.3	136.538.875	140.089.907	5	MIR3689A, MIR3689B, MIR126, MIR4292, MIR3621
R337H+	10q21.3 - q26.3	64.967.583	134.372.883	18	MIR1296, MIR1256, MIR346, MIR107, MIR3157, MIR1287, MIR608, MIR3158-1, MIR3158-2, MIR146B, MIR1307, MIR936, MIR609, MIR4295, MIR2110, MIR3941, MIR4297, MIR378C
R337H+	10q26.3	134.427.068	135.101.326	1	MIR202
R337H+, R337H-	11p15.5	353.347	3.011.146	4	MIR210, MIR4298, MIR675, MIR483
R337H+	11q12.2-q14.1	61.582.542	78.277.811	9	MIR1908, MIR192, MIR194-2, MIR612, MIR548K, MIR3664, MIR3165, MIR139, MIR326
R337H-	11q13.3-q13.4	69.422.459	70.647.377	1	MIR548K
R337H+	11q13.4 - q25	73.249.469	134.868.407	14	MIR326, MIR708, MIR4300, MIR1260B, MIR3920, MIR34B, MIR34C, MIR4301, MIR3656, MIR100HG, MIR125B1, MIRLET7A2, MIR100, MIR3167
R337H+	12q12 - q24.33	38.645.610	133.707.336	30	MIR1291, MIR1293, MIR196A2, MIR615, MIR148B, MIR1228, MIR26A2, MIRLET7I, MIR548C, MIR548Z, MIR1279, MIR3913-1, MIR3913-2, MIR1252, MIR617, MIR618, MIR492, MIR331, MIR3685, MIR1251, MIR135A2, MIR4303, MIR1827, MIR3652, MIR3922,MIR620, MIR1178, MIR4304, MIR3908, MIR3612
R337H+	13q11 - q34	19.296.544	114.581.677	23	MIR4306, MIR623, MIR3170, MIR17HG, MIR17, MIR18A, MIR19A, MIR20A, MIR19B1, MIR92A1, MIR622, MIR3665, MIR3169, MIR1297, MIR759, MIR16-1, MIR15A, MIR3613, MIR621, MIR320D1, MIR4305, MIR548F5, MIR2276
R337H+	13q34	114.528.643	114.886.865	0	

Cont. Table S1.

Groups of patients	Cytoband	Start	Stop	#miRNAs	miRNA
R337H+	14q21.1 - q32.33	38.168.366	107.258.824	58	MIR379, MIR411, MIR299, MIR380, MIR1197, MIR323, MIR758, MIR329-1, MIR329-2, MIR494, MIR1193, MIR543, MIR495, MIR376C, MIR376A2, MIR654, MIR376B, MIR376A1, MIR300, MIR1185-1, MIR1185-2, MIR381, MIR487B, MIR539, MIR889, MIR655, MIR487A, MIR382, MIR134, MIR668, MIR485, MIR323B, MIR154, MIR496, MIR377, MIR541, MIR409, MIR412, MIR369, MIR410, MIR656, MIR203, MIR4309, MIR1247, MIR431, MIR433, MIR127, MIR432, MIR136, MIR770, MIR493, MIR337, MIR665, MIR345, MIR3173, MIR1260, MIR4308, MIR548Y
R337H+, R337H-	14q32.33*	105.052.722	106.957.950	0	
R337H+, R337H-	16p13.3-p11.2	106.271	32.624.637	22	MIR762, MIR548D2, MIR548AA2, MIR484, MIR3180-4, MIR3179-1, MIR3179-2, MIR3179-3, MIR3180-1, MIR3180-3, MIR3180-2, MIR193B, MIR365-1, MIR548X, MIR3178, MIR3677, MIR940, MIR1225, MIR3180-5, MIR3177, MIR662, MIR3176
R337H+	16q11.2-q24.3	46.564.557	90.111.263	10	MIR1910, MIR3182, MIR3647, MIR1972-1, MIR1972-2, MIR140, MIR1538, MIR328, MIR138-2, MIR3935
R337H+	16q24.2-q24.3	88.506.907	89.712.281	0	
R337H+, R337H-	17p13.3 - p11.2	87.009	20.649.165	13	MIR3183, MIR33B, MIR1288, MIR744, MIR3676, MIR4314, MIR195, MIR497, MIR324, MIR1253, MIR132, MIR212, MIR22
R337H+	17q11.1 - q25.3	25.636.226	81.029.941	32	MIR3186, MIR657, MIR3065, MIR338, MIR1250, MIR4316, MIR636, MIR3678, MIR3615, MIR635, MIR634, MIR548W, MIR21, MIR454, MIR301A, MIR142, MIR3185, MIR196A1, MIR10A, MIR1203, MIR152, MIR4315-1, MIR4315-2, MIR2117, MIR2909, MIR632, MIR193A, MIR365-2, MIR423, MIR3184, MIR451, MIR144
R337H+, R337H-	17q25.3	76.334.944	81.029.941	5	MIR657, MIR3065, MIR338, MIR1250, MIR3186
R337H+	18q11.1-q12.3	18.620.055	42.686.197	8	MIR4319, MIR4318, MIR187, MIR302F, MIR320C2, MIR133A1, MIR1-2, MIR320C1
R337H+	19p13.11	18888734	18963985	0	
R337H+	19p13.2	7.554.235	10.559.626	3	MIR4322, MIR941-3, MIR941-2
R337H+	19p13.3	3.587.115	6.797.381	4	MIR637, MIR7-3, MIR3940, MIR1181
R337H+	19q13.2 - q13.33	38.896.379	48.879.628	9	MIR3190, MIR3191, MIR320E, MIR769, MIR642A, MIR642B, MIR330, MIR4323, MIR641

Cont. Table S1.

Groups of patients	Cytoband	Start	Stop	#miRNAs	miRNA
R337H+	19q13.33 - q13.43	49.593.920	59.077.930	55	MIR512-2, MIR512-1, MIR1323, MIR498, MIR520E, MIR515-1, MIR515-2, MIR519E, MIR520F, MIR519C, MIR1283-1, MIR520A, MIR526B, MIR519B, MIR525, MIR523, MIR518F, MIR520B, MIR518B, MIR526A1, MIR520C, MIR518C, MIR524, MIR517A, MIR519D, MIR521-2, MIR520D, MIR517B, MIR520G, MIR516B2, MIR526A2, MIR518E, MIR518A1, MIR518D, MIR516B1, MIR518A2, MIR517C, MIR520H, MIR521-1, MIR522, MIR519A1, MIR527, MIR516A1, MIR1283-2, MIR516A2, MIR519A2, MIR371, MIR372, MIR373, MIR99B, MIRLET7E, MIR125A, MIR643, MIR4324, MIR150
R337H+	20q11.21	30.193.614	30.309.568	1	MIR3193
R337H+, R337H-	20q13.31 - q13.33	55.745.834	62.893.189	14	MIR941-1, MIR941-3, MIR941-2, MIR1914, MIR647, MIR4326, MIR3196, MIR124-3, MIR1-1, MIR133A2, MIR1257, MIR296, MIR298, MIR4325
R337H+	20q13.33	59602175	60575265	1	MIR1257
R337H+	20q13.33	60575206	62336155	5	MIR1-1, MIR133A2, MIR3196, MIR124-3, MIR4326
R337H+, R337H-	21q22.3	46907794	47003531	0	
R337H+	22q11.1 - q13.33	17280847	51178264	25	MIR3198, MIRLET7A3, MIRLET7B, MIR3201, MIR1249, MIR3619, MIR33A, MIR1281, MIR658, MIR659, MIR3909, MIR3199-1, MIR3199-2, MIR3200, MIR3928, MIR3653, MIR548J, MIR650, MIR301B, MIR130B, MIR1286, MIR1306, MIR3618, MIR185, MIR648
R337H+, R337H-	22q11.21	20.217.132	20.270.498	1	MIR1286
R337H+, R337H-	22q11.22*	23056562	23190273	1	MIR650
R337H+	22q13.31	46.316.673	46.547.478	0	
R337H+	Xq28	152660883	152937101	0	

*indicate cytobands that were presented with CNAs in >50% of the R337H+ and/or R337H- group of patients.

Table S2. Genes observed in the R337H+ and R337H- groups of patients upon integration of the genes located at the cytobands most affected by CNAs and the miRNA target genes mapped at the same cytobands and their association with survival in the KMplot database.

Groups of patients	Genes	Chrom.	Breast cancer cases					
			All samples		TP53 mutation		TP53 wild type	
			HR	P value	HR	P value	HR	P value
	ECM1	1q21.2	0.88 (0.79-0.98)	0.0195	1.87 (1.14-3.08)	0.012	0.98 (0.64- 1.49)	0.92
	MCL1	1q21.2	0.85 (0.76- 0.95)	0.0033	0.45 (0.28- .77)	0.0021	0.72 (0.47- 1.1)	0.19
	RORC	1q21.3	0.6 (0.54- 0.67)	<1e-16	1.01 (0.63- 1.63)	0.96	1.1 (0.72- 1.67)	0.67
	PEA15	1q23.2	1.07 (0.96-1.19)	0.2312	0.85 (0.53- 1.37)	0.51	1.01 (0.66-1.53)	0.98
	RGS4	1q23.3	0.96 (0.86-1.07)	0.4265	1.62 (0.99-2.63)	0.051	1.24 (0.81- 1.89)	0.32
	PRRX1	1q24.2	0.79 (0.71-0.88)	2.4e-5	0.98(0.61-1.58)	0.94	0.71 (0.47-1.09)	0.11
	RASAL2	1q25.2	1.32 (1.19- 1.48)	4.2e-7	0.9 (0.56- 1.45)	0.67	0.74 (0.49-1.13)	0.17
	LAMC1	1q25.3	1.12 (1- 1.24)	0.0492	1.5 (0.93-2.42)	0.09	1.19 (0.78-1.81)	0.42
	PTGS2	1q31.1	0.84 (0.75- 0.94)	0.0019	0.9(0.56-1.45)	0.67	0.82 (0.54-1.25)	0.36
	TRAF5	1q32.3	NF	NF	NF	NF	NF	NF
	WNT3A	1q42.13	NF	NF	NF	NF	NF	NF
	FH	1q43	1.36 (1.22- 1.51)	4.4e-8	0.82 (0.51-1.33)	0.43	1.05 (0.69-1.6)	0.81
	SMYD3	1q44	1.05 (0.94- 1.17)	0.3825	2.37 (1.44-3.89)	0.00044	1.21 (0.79-1.85)	0.38
	MAP4K4	2q11.2	1.36 (1.22- 1.52)	2.4e-8	1.04 (0.65-1.66)	0.88	0.98(0.65-1.5)	0.94
	LIMS1	2q12.3	1.27 (1.14- 1.41)	1.9e-5	1.23 (0.77-1.98)	0.39	0.75 (0.49-1.15)	0.19
	BUB1	2q13	1.79 (1.6-2)	<1e-16	0.77 (0.48-1.25)	0.29	1.93 (1.25-2.97)	0.0024
	BCL2L11	2q13	0.6 (0.51-0.7)	2.1e-10	0.72 (0.4-1.3)	0.28	1.37 (0.58-3.19)	0.47
	HAT1	2q31.1	1.22 (1.09-1.36)	0.00035	0.97(0.6-1.56)	0.9	0.7 (0.45-1.07)	0.098
	ITGA6	2q31.1	0.89 (0.8-1)	0.0407	0.88 (0.55-1.41)	0.6	0.55 (0.35-0.85)	0.0064
R337H+	***HOXD10***	2q31.1	0.68 (0.58- 0.8)	1.2e-6	0.59 (0.32-1.07)	0.077	2.39 (0.98-5.82)	0.049
	STAT1	2q32.2	0.85 (0.77-0.95)	0.0045	0.39 (0.23-0.65)	0.00019	0.69 (0.45-1.06)	0.088
	NRP2	2q33.3	0.92 (0.82-1.02)	0.13	1.16 (0.72-1.86)	0.55	1.09(0.71- 1.65)	0.7
	CREB1	2q33.3	0.87 (0.78-0.98)	0.016	1.1(0.69-1.77)	0.69	0.82 (0.53-1.24)	0.34
	FN1	2q35	1.1 (0.98-1.22)	0.095	1.21 (0.75-1.96)	0.42	0.87 (0.57-1.32)	0.51
	IGFBP5	2q35	1.02 (0.91- 1.13)	0.76	1.8(1.1-2.94)	0.018	0.95(0.62-1.44)	0.8
	CTDSP1	2q35	0.65 (0.58- 0.73)	1.4e-14	1.47(0.91-2.39)	0.12	1.24 (0.81-1.89)	0.32
	DNPEP	2q35	0.69 (0.62- 0.77)	2.6e-11	0.83(0.51-1.33)	0.43	0.84(0.55-1.29)	0.43
	HDAC4	2q37.3	0.76 (0.65- 0.89)	0.00069	0.78(0.43-1.4)	0.39	1.82(0.78-4.28)	0.16
	SEPT2	2q37.3	1.13 (1.02- 1.26)	0.025	0.69(0.43-1.11)	0.13	0.73(0.48-1.12)	0.14
	GPC1	2q37.3	0.83 (0.74- 0.92)	0.00055	1.25(0.78-2.02)	0.35	1.19(0.78-1.81)	0.43
	ADAM9	8p11.22	0.92 (079-1.07)	0.28	1.01 (0.63-1.62)	0.97	0.44(0.18-1.07)	0.065
	CCNE2*	8q22.1	1.97 (1.76- 2.21)	<1e-16	1.37 (0.85- 2.2)	0.19	1.32 (0.87-2.02)	0.19
	*SNAI2**	8q11.21	1.06 (0.95- 1.18)	0.2975	0.84 (0.55- 1.28)	0.42	0.84 (0.55-1.28)	0.42
	RDH10*	8q21.11	1.31 (1.31- 1.53)	0.0005	1.33 (0.74-2.39)	0.34	0.66 (0.28- 1.55)	0.34
	BNIP3L	8q21.2	0.89 (0.8- 0.99)	0.037	1.06 (0.66-1.7)	0.82	0.68 (0.44-1.04)	0.073
	MMP16	8q21.3	0.83 (0.75- 0.93)	0.0009	1.68(1.03-2.72)	0.035	0.9(0.59-1.38)	0.64
	MTDH*	8q22.1	1.2 (1.07- 1.33)	0.0012	1.54 (0.85- 2.78)	0.15	0.91 (0.39-2.09)	0.82
	FZD6	8q22.3	1.26 (1.13- 1.4)	3.6e-5	1.33(0.83-2.14	0.24	0,92(0.6-1.4)	0.68

Cont. Table S2.

	Genes	Chrom.	Breast cancer cases					
			**All samples**		**TP53 mutation**		**TP53 wild type**	
			**HR**	**P value**	**HR**	**P value**	**HR**	**P value**
	CTHRC1	8q22.3	1.19 (1.02- 1.39)	0.026	1.92 (1.05-3.51)	0.031	0.66(0.28-1.55)	0.34
	HAS2	8q24.13	0.91 (0.81- 1.01)	0.079	1.26-(0.78-2.03)	0.34	0.81 (0.53-1.24)	0.33
	MYC	8q24.21	1.12 (1- 1.24)	0.049	0.8 (0.5-1.29)	0.36	0.83 (0.54-1.26)	0.37
	MAF	16q23.2	0.98 (0.88- 1.09)	0.73	0.57 (0.35-1.93)	0.022	0.98 (0.64-1.49)	0.92
	FASN	17q25.3	0.82 (0.74- 0.91)	0.00035	1.28 (0.79-2.06)	0.31	1.59 (1.04-2.44)	0.032
**R337H-**	***OXR1***	8q23.1	1.28 (1.14-1.42)	1.1e-5	1.09 (0.68-1.76)	0.71	0.62 (0.4- 0.95)	0.027
	ZFPM2	8q23	0.81 (0.73-0.91)	0.00021	0.85 (0.53-1.37)	0.52	0.81 (0.53- 1.24)	0.33
	SOX17	8q11.23	0.72 (0.69-0.8)	3.6e-9	1.54 (0.95-2.5)	0.079	0.84 (0.55- 1.28)	0.42

In bold: genes which expression showed to be correlated with survival (both in the general BrCa cases of the KMPlot and in cases based on TP53 mutations); * genes commonly observed in the R337H+ and R337H- group of patients; HR:Hazard ratio; HR:1 no correlation; HR>1, higher risk of death; HR<1 lower risk of death; NF: not found
